# Traumatic Ankle Injuries in the Emergency Department: Evaluating the Adequacy of Clinical Documentation With Reference to the Ottawa Ankle Rules

**DOI:** 10.7759/cureus.95645

**Published:** 2025-10-29

**Authors:** Amna Anwar, Muhammad H Ali, Haram Ali, Walid Anwar, Mohamed S Baalawi

**Affiliations:** 1 Medicine and Surgery, Queen's Hospital/King George's Hospital, London, GBR; 2 Radiology, Barts Health NHS Trust, London, GBR; 3 Medicine and Surgery, University of Leicester, Leicester, GBR; 4 Surgery, Homerton University Hospital, London, GBR; 5 Emergency Medicine, Barts Health NHS Trust, London, GBR

**Keywords:** ankle trauma, clinical decision rules, fracture detection, joint imaging, musculoskeletal radiology, ottawa ankle rules, quality improvement projects, sports injuries, x-ray radiography

## Abstract

Introduction

Ankle trauma is a frequent presentation to the emergency department (ED) and poses a significant demand on radiology services. Efficient triage is essential to identify patients requiring urgent imaging, optimising resources, reducing waiting times, and minimising unnecessary radiation exposure. Injuries range from soft tissue damage to fractures of the distal tibia, fibula, or malleoli. Not all ankle injuries require immediate imaging, and clinical decision-making tools can guide the use of radiographs. The Ottawa Ankle Rules (OAR) are a validated tool, endorsed by the Royal College of Radiologists (RCR), designed to identify ankle injuries requiring radiographs. Imaging is indicated if the patient is unable to bear weight both immediately after injury and during examination, or if there is bone tenderness along the distal six centimetres of the posterior edge of the tibia or fibula, or over the lateral or medial malleoli. Adherence to the OAR has been shown to reduce unnecessary imaging while maintaining high sensitivity for fractures.

Methods

A two-cycle retrospective review was conducted of patients presenting with traumatic ankle injuries who underwent radiography. Records were reviewed via the Picture Archiving and Communication System (PACS) until 100 patients per cycle were included. Data collected included whether the OAR were referenced in radiograph requests and whether a fracture was diagnosed. After the first cycle, educational interventions were implemented to increase guideline awareness. The second cycle evaluated the impact of these interventions. Statistical analysis assessed the significance of observed changes. Exclusions included patients aged ≤16 years or ≥55 years, those with non-traumatic or chronic ankle presentations, and those with polytrauma or high-energy mechanisms of injury. Results were compared against RCR standards, which state that 100% of all ankle plain-film requests for trauma should reference the OAR. Results were presented at departmental governance meetings, and recommendations were subsequently implemented.

Results

In the first cycle, 37 (37%) of radiograph requests referenced the OAR. Fractures were identified in 21 (21%) patients, with 13 (61.9%) of these requests documenting the OAR and 8 (38.1%) omitting them. Following educational interventions, the second cycle demonstrated significant improvement, with 51 (51%) of requests referencing the OAR (P = 0.046). The proportion of patients with fractures increased to 31 (31%), of whom 26 (83.9%) had OAR documented, compared with 5 (16.1%) without documentation (P < 0.001). These findings reinforce that adherence to the OAR improves fracture detection, reduces unnecessary imaging, and remains a sensitive tool for ankle trauma assessment.

Conclusion

This quality improvement project demonstrates that focused interventions, particularly clinician education and guideline awareness, can enhance adherence to the OAR. Increased documentation and use of the OAR were associated with a higher diagnostic yield for fractures, improved triage, and a potential reduction in unnecessary imaging. Although full compliance was not achieved, incremental improvements contribute to patient safety and alignment with national recommendations. Future strategies may include regular departmental teaching sessions and system-based interventions such as electronic prompts to sustain compliance.

## Introduction

Ankle trauma represents one of the most frequent presentations to the emergency department (ED), with sequelae ranging from minor ligamentous injuries to significant fractures requiring urgent intervention. Data suggest that ankle injuries account for up to 5% of all ED presentations, whereas less than 15% of all radiographs are positive for a fracture [1,2]. Diagnostic imaging plays a crucial role in evaluating these injuries; with increasing patient volumes and pressures on radiology services, it is important to ensure judicious use of radiographs. The Royal College of Radiologists (RCR) recommends that all imaging requests be justified using evidence-based guidelines such as the Ottawa Ankle Rules (OAR) to ensure appropriate triaging of cases, efficient use of radiological services, and reduced unnecessary radiation exposure [3].

The OAR recommend that radiographs should only be obtained in patients presenting with ankle trauma if they demonstrate inability to bear weight (walk four steps) immediately after the injury and during examination in the ED, or if there is bone tenderness along the distal six centimetres of the posterior edge of the fibula or tibia, or over the lateral or medial malleoli [4]. Since their development, these rules have been shown to provide excellent sensitivity for detecting clinically significant fractures while also reducing the number of unnecessary radiographs performed [2,4]. Implementation of the OAR has been shown to significantly reduce unnecessary radiographic imaging, thereby decreasing healthcare costs, minimising patient exposure to radiation, and improving efficiency within emergency department settings [5].

Despite strong evidence for their use, adherence to the OAR is variable and not universal across emergency departments. In some cases, omission of appropriate clinical detail or referencing of these rules in radiology requests may undermine proper justification under the Ionising Radiation (Medical Exposure) Regulations (IR(ME)R), which require that imaging be supported by sufficient clinical information (RCR iRefer) [3]. Audits and quality improvement projects addressing OAR implementation have shown that targeted interventions, such as clinician education and standardised request formats, can improve compliance, optimise patient care, and ensure more efficient allocation of healthcare resources [6].

Our study aimed to evaluate clinician compliance with the OAR when requesting ankle radiographs for traumatic injuries at a district general hospital in East London. A two-cycle audit was conducted to assess baseline practice, improve compliance through education-based interventions, and measure subsequent improvement in documentation and adherence.

## Materials and methods

Ethics statement

Prior to commencement, formal approval was obtained from the local Clinical Audit Department at Newham University Hospital to ensure ethical and methodological integrity.

Data collection was conducted in accordance with institutional and national governance standards, following the NHS Digital Anonymisation Standard for Publishing Health and Social Care Data (ISB1523) and the Information Commissioner’s Office (ICO) guidance to ensure patient confidentiality [7,8]. Data were securely stored on encrypted hospital systems accessible only to authorised members of the audit team.

Study design

This study represents a comprehensive retrospective analysis of ankle plain films conducted at Newham University Hospital, investigating compliance with RCR guidance pertaining to the OAR for imaging requests in cases of ankle trauma. The audit was undertaken as a two-cycle quality improvement study within the framework of the Plan-Do-Study-Act (PDSA) model, designed to assess baseline performance, implement targeted interventions, and evaluate subsequent improvement [9].

The first audit cycle spanned August to September 2024, while the second cycle was undertaken between October and November 2024, following the implementation of educational interventions. Both cycles adhered to identical methodologies, allowing for direct comparison of outcomes and minimising confounding variables.

Study population and sample size

The study population included adult patients presenting with acute ankle trauma who underwent ankle plain-film imaging during the defined audit periods. A target sample size of 100 patients per cycle was determined to provide a representative cohort sufficient for statistical comparison. Participant selection for both cycles was undertaken by the same authors to minimise observer bias.

Exclusion criteria included adults aged 55 years or older, as some studies suggest reduced specificity of the OAR in this cohort. Similarly, patients aged 16 years or younger were excluded, as paediatric-specific rules apply in such cases [5,10]. Plain films obtained for patients presenting with non-traumatic ankle conditions were not included, as the OAR are only validated in cases of ankle trauma. In addition, patients with polytrauma or high-energy mechanisms of injury were excluded, as these patients are at higher risk of complex injuries for which plain radiography guided by the OAR may be insufficient.

In the first audit cycle, a total of 291 ankle radiographs were reviewed. Of these, the following were excluded according to the criteria described above: 88 cases with non-traumatic indications (including infection, investigation of chronic pain, and orthopaedic follow-up scans); 24 patients aged 16 years or younger; 74 patients aged 55 years or older; and 5 cases involving polytrauma or high-energy mechanisms of injury (Table 1).

**Table 1:** Exclusion of Patients During the First Audit Cycle (n = 291)

Exclusion Criteria	Number Excluded (n)	Percentage of Total (%)
Other indications (non-trauma)	88	30.2
Age ≤ 16 years	24	8.2
Age ≥ 55 years	74	25.4
Polytrauma/high-energy mechanism	5	1.7
Total excluded	191	65.6
Total included for analysis	100	34.4

In the second audit cycle, a total of 365 ankle radiographs were reviewed. Exclusions comprised 91 cases with non-traumatic indications, 61 patients aged 16 years or younger, 100 patients aged 55 years or older, and 13 cases involving polytrauma or high-energy injury mechanisms (Table 2).

**Table 2:** Exclusion of Patients During the Second Audit Cycle (n = 365)

Exclusion Criteria	Number Excluded (n)	Percentage of Total (%)
Other indications (non-trauma)	91	24.9
Age ≤ 16 years	61	16.7
Age ≥ 55 years	100	27.4
Polytrauma/high-energy mechanism	13	3.6
Total excluded	265	72.6
Total included for analysis	100	27.4

Plain films were reviewed and excluded until 100 eligible patients were included in each audit cycle for analysis. The higher number of exclusions in the second cycle largely reflected an increased volume of imaging requests over the audit period, particularly within the paediatric population. Applying identical exclusion parameters across both cycles ensured direct comparability and reduced the potential influence of confounding variables such as demographic or clinical differences between cohorts.

Study measures

Data collection was performed using the hospital’s Electronic Patient Record (EPR) system, Cerner Millennium, and the Picture Archiving and Communication System (PACS). For each case, data collected included patient demographics (age and sex), the clinical indication for imaging with explicit reference to the OAR within the imaging request, and the final radiographic outcome indicating whether a fracture was identified.

Compliance was assessed against the RCR standard, which specifies that 100% of all ankle plain-film requests for trauma should reference the OAR [3]. Following completion of the first audit cycle, findings were presented at clinical governance meetings, where recommendations to improve compliance were developed and implemented. Interventions included focused education sessions for front-line clinicians, as well as targeted briefings for radiographers, who are the primary personnel involved in vetting X-ray requests. Emphasis was placed on the importance of performing a clinical examination in accordance with the OAR and ensuring that these findings were documented within imaging requests to support an appropriate vetting process.

A second audit cycle was conducted following an appropriate implementation period for these interventions to determine their effects.

Statistical analysis

All data were collated and analysed using Microsoft Excel (Microsoft Corporation, Redmond, Washington). Descriptive statistics were employed to summarise patient demographics and compliance rates. Comparisons between audit cycles were assessed using the two-proportion z-test to determine changes in documentation compliance with the OAR. The chi-square test was used to evaluate the association between positive radiographic findings (presence of fracture) and inclusion of OAR documentation in imaging requests. A P-value < 0.05 was considered statistically significant.

## Results

Table 3 provides an overview of the data collected in both audit cycles, including the number of patients reviewed, the proportion of requests referencing the OAR, and the fracture detection rate. 

**Table 3:** Patient Numbers and Key Results Across Both Audit Cycles Data are expressed as number of patients (N) and percentage (%). P-value significant at <0.05. OAR: Ottawa Ankle Rules.

Variables/Measure	First Cycle	Second Cycle	P-value
Gender – Male	52 (52.0%)	55 (55.0%)	-
Gender – Female	48 (48.0%)	45 (45.0%)	-
Age range (N)	18–54	17–54	-
Mean age (SD)	31.5 (10.2)	32.26 (9.22)	-
Requests referencing OAR	37 (37.0%)	51 (51.0%)	0.046
Fractures diagnosed	21 (21.0%)	31 (31.0%)	-
Fractures with OAR referenced	13 (61.9%)	26 (83.9%)	-
Fractures without OAR referenced	8 (38.1%)	5 (16.1%)	-

In the first audit, the sample comprised 52 (52.0%) males and 48 (48.0%) females. Ages ranged from 18 to 54 years, with a mean age of 31.5 years and a standard deviation of 10.2. In the second audit, ages ranged from 17 to 54 years, with a mean of 32.26 years and a standard deviation of 9.22. Of these, 55 (55.0%) were male and 45 (45.0%) were female, indicating that patient gender and age were consistent across both cycles.

In the first cycle of the study, 100 imaging requests for patients presenting to the emergency department with ankle trauma were reviewed. Of these, 37 (37.0%) requests explicitly documented application of the OAR. This fell significantly below the target of 100% set by the RCR. Additionally, a total of 21 (21.0%) patients were subsequently diagnosed with a fracture. Among these, 13 (61.9%) patients had imaging requests that referenced the OAR, while the remaining 8 (38.1%) did not. This difference was statistically significant (P = 0.011), supporting existing evidence that use of the OAR is an effective, evidence-based approach for triaging ankle trauma and identifying cases associated with fracture.

**Figure 1:**
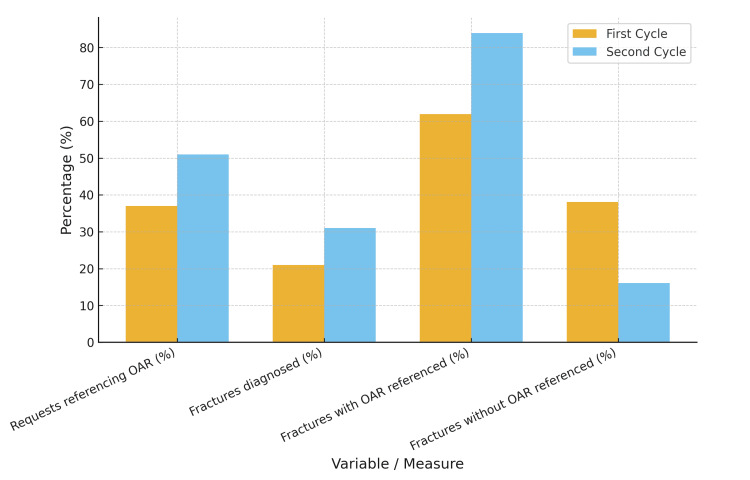
Comparison of Audit Cycles: Ottawa Ankle Rules (OAR) Compliance and Outcomes The figure compares the first and second audit cycles across key measures.

Following a period of targeted education and departmental reinforcement of guidelines, a second audit cycle was undertaken. Documentation of OAR compliance improved significantly, increasing from 37 (37.0%) in the first cycle to 51 (51.0%) in the second (P = 0.046). This demonstrates that educational interventions can enhance compliance with guidelines, although further work remains to achieve the 100% target set by the RCR, as illustrated in Figure 1, which compares the first and second audit cycles across key measures of OAR compliance and fracture outcomes.

Additionally, in this cohort, 31 (31.0%) patients were diagnosed with a fracture, compared with 21 (21.0%) in the first cycle. Of those with confirmed fractures, 26 (83.9%) requests referenced the OAR, whereas only 5 (16.1%) did not, a difference that was highly significant (P < 0.001). These findings again reinforce the clinical value of OAR implementation in the assessment of ankle trauma.

## Discussion

In this quality improvement project, we aimed to align clinical practice with evidence-based standards, as outlined in RCR guidance.

The OAR were first introduced in 1992 to guide the use of radiography in acute ankle injuries [4]. Subsequent implementation studies confirmed that these rules could be applied safely in emergency settings, reducing unnecessary imaging while maintaining high sensitivity for detecting clinically significant fractures [1]. Systematic reviews have since reinforced the reliability of the rules, consistently demonstrating high sensitivity in ruling out ankle fractures [2]. Missing a fracture may lead to complications such as compartment syndrome, non-union, and early-onset osteoarthritis. Appropriate triage and assessment can help avoid delays and reduce the risk of such adverse outcomes [11].

Beyond improving diagnostic accuracy and avoiding complications, adherence to the OAR has broader implications for patient safety and healthcare delivery. Implementing the OAR as a care protocol for acute ankle sprains has been shown to be feasible and effective in reducing unnecessary radiographs [12]. Although ankle radiographs involve relatively low radiation doses, the Ionising Radiation (Medical Exposure) Regulations (IR(ME)R) stipulate that all imaging must be clinically justified and supported by adequate clinical information [3]. Minimising unnecessary radiographs reduces patient radiation exposure, eases pressure on radiology departments, shortens waiting times, and helps prioritise patients requiring urgent medical attention. By addressing each of these factors, the use of the OAR ultimately contributes to the overarching goal of reducing patient harm.

A key finding from this audit is that adherence to the OAR improves both the appropriateness and diagnostic value of imaging requests. Requests that explicitly included OAR documentation were associated with a significantly higher fracture detection rate compared with those without, highlighting the utility of these rules in improving the diagnostic yield of radiography and prioritising imaging for patients at higher risk of clinically significant injury [2,4].

Despite the strong evidence base, our first-cycle findings revealed suboptimal adherence in routine clinical practice. To address this, we implemented a multifaceted educational intervention, initially targeting radiographers as the primary personnel involved in vetting ankle plain films, and later expanding to include frontline healthcare professionals assessing injuries [13]. Our two-cycle audit showed that relatively simple interventions, such as presenting at governance meetings and organising teaching sessions as awareness initiatives, can meaningfully improve guideline adherence, consistent with other studies [13]. A study conducted at Lincoln County Hospital demonstrated that a two-cycle audit, incorporating educational interventions such as distributing handouts and delivering teaching sessions, led to a significant improvement in compliance with the Ottawa Foot and Ankle Rules [14]. However, despite this improvement, a significant deficit remained in achieving the 100% target set by the RCR, which will need to be addressed in future cycles. Despite improvements in compliance, challenges remain in achieving 100% adherence to the OAR, indicating the need for ongoing education and system-level interventions [13].

One way to improve adherence going forward could be to embed OAR prompts within electronic radiology request systems, which would be especially valuable given the frequent rotation of junior doctors in emergency departments. A multifaceted strategy, including educational meetings, reminder posters, and clinical decision-support systems, has been effective in increasing adherence to the OAR and reducing unnecessary imaging [15]. Similarly, incorporating educational measures into induction programmes for each new cohort would help maintain ongoing awareness of the guidelines among all staff. Follow-up audits after these interventions could then evaluate whether enhanced clinical documentation translates into a greater proportion of positive radiographs and more effective prioritisation of high-risk patients.

This audit has several limitations. Its retrospective design meant that the analysis depended on the quality of existing documentation; it is possible that clinicians applied the OAR in practice without formally recording it. Additionally, the single-centre setting limits the generalisability of the findings. Future work could involve multicentre audits encompassing other hospitals within the trust.

## Conclusions

This quality improvement project demonstrated that focused interventions, particularly clinician education and increased awareness of clinical guidelines, can significantly enhance adherence to the use of the OAR as an evidence-based tool for assessing ankle trauma. By promoting the documentation and application of the OAR in radiograph requests, we observed a marked improvement in compliance and an associated increase in the diagnostic yield of imaging for fractures. These improvements are likely to contribute to better patient outcomes by enabling accurate triage, reducing complication rates, and avoiding unnecessary radiation exposure.

Although complete compliance with documentation standards was not achieved, each step forward supports the overarching goal of improving patient safety and aligning clinical decisions with national recommendations. Future studies will aim to assess the impact of regular departmental teaching sessions for rotating clinicians and passive system-based interventions, such as electronic prompts.
